# Impulsivity and Reasons for Living Among African American Youth: A Risk-Protection Framework of Suicidal Ideation

**DOI:** 10.3390/ijerph120505196

**Published:** 2015-05-15

**Authors:** Temilola K. Salami, Bianca A. Brooks, Dorian A. Lamis

**Affiliations:** 1 Department of Clinical Psychology, University of Georgia, 125 Baldwin St., Athens, GA 30602, USA; E-Mail: temisalami@gmail.com; 2 Department of Clinical Psychology, Georgia State University, 140 Decatur St., Atlanta, GA 30303, USA; E-Mail: bbrooks11@student.gsu.edu; 3 Department of Psychiatry and Behavioral Sciences, School of Medicine, Emory University, 80 Jesse Hill Jr. Drive, Atlanta, GA 30303, USA

**Keywords:** reasons for living, suicide, impulsivity, African American

## Abstract

This study aims to explore the impact of specific facets of impulsivity as measured by the UPPS Impulsive Behavior Scale (UPPS), as well as reasons for living in predicting suicidal ideation among African American college-aged students. The incremental validity of each facet of the UPPS interacting with reasons for living, a construct meant to buffer against risk for suicide, was explored in a sample of African American students (*N* = 130; ages 18–24). Results revealed significant interactions between reasons for living and two factors of impulsivity, (lack of) premeditation and sensation seeking. Higher levels of sensation seeking and lack of premeditation in conjunction with lower reasons for living was associated with increased suicidal ideation. Neither urgency nor (lack of) perseverance significantly interacted with reasons for living in association with suicidal ideation. These results suggest including elements of impulsivity, specifically sensation seeking and (lack of) premeditation, when screening for suicidal ideation among African American youth. Future investigations should continue to integrate factors of both risk and protection when determining risk for suicide.

## 1. Introduction

Although African Americans have traditionally had lower rates of suicide than other ethnic groups [1], suicide remains the third leading cause of death among African American youth [2,3]. Furthermore, African Americans may not readily seek formal help for suicidal ideation [4] and may not readily disclose suicidal thoughts once help is sought [5]. Researchers have documented the potential reasons for reduced help seeking among African Americans, which include fear of stigma, mistrust of mental health services and paraprofessionals, and possible embarrassment [6,7,8]. Given the limited report of suicidal ideation and help seeking among individuals in the African American community, it is not surprising that the prevailing knowledge on risk and protective factors for African Americans who experience suicidal ideation is scarce. However, given that suicide remains a leading cause of death for African American youth [2], it is imperative that attention be devoted to understanding factors that contribute to the experience of suicidality using a framework that assesses both risk and protective factors. Risk-protection models provide a more comprehensive view of the course and maintenance of psychopathology (see [9,10,11]). Thus, we employ a risk-protection framework in the current study.

There is a growing body of literature concerning impulsivity as a risk factor for suicidality, with studies showing that higher levels of impulsivity are associated with an increased risk for suicidality (e.g., [12,13]). Furthermore, previous investigations have noted that African Americans may have culturally salient variables that help protect against risk of suicide (e.g., [14,15,16,17,18]). Reasons for living may be particularly relevant among African Americans and may help buffer suicide risk. African Americans report higher levels of reasons for living than European Americans, and reasons for living has been found to be associated with other culturally relevant protective variables (e.g., religiosity and communal support) for African Americans (see [19]). Therefore, in the current study, we examined the protective role of reasons for living and risk of impulsivity on self-reported suicidal ideation among African American youth.

### 1.1. The Differential Role of Impulsivity: Risk

The construct of impulsivity has been explored as a pathway for psychopathology [20], as well as suicidality [12,21]. Suicide is often considered an impulsive act and difficulty controlling aggressive impulses has been associated with a higher likelihood of impulsive suicide attempts than depression alone [22]. Although research suggests a positive relation between impulsivity and suicidality, this connection in African Americans has received little attention. One study by Kaslow and colleagues [23] found that African Americans who had attempted suicide previously endorsed higher levels of impulsivity as compared to non-attempters. Given the association between impulsivity and suicidal outcomes among African Americans, further attention needs to be paid to the understanding of the types of impulsive actions that are important to the onset and maintenance of suicidality among African American youth.

The operationalization of impulsivity is quite heterogeneous and the facets within impulsivity that lead to suicidal beliefs have not been fully delineated. Understanding what facets of impulsivity are most important in their association with suicidal ideation will provide future investigators and practitioners with greater understanding and greater predictive utility for suicidal outcomes. A common tool that has been used within the literature to assess impulsivity is the UPPS Impulsive Behavior Scale (UPPS). Developed by Whiteside and Lynam [20], the UPPS includes four related yet distinct individual difference personality facets to define impulsive behavior: urgency, sensation seeking, (lack of) premeditation, and (lack of) perseverance. Urgency refers to one’s propensity to experience and act on strong impulses; (lack of) premeditation assesses a person’s inability to anticipate consequences prior to engaging in a certain behavior; (lack of) perseverance pertains to a person’s ability to persist on tasks that may be boring or challenging; and sensation seeking refers to both an openness to try new experiences that may or may not be dangerous, and proclivity for activities that are exciting [20].

The differential validity of the four facets of the UPPS has been explored as it pertains to a variety of clinical disorders [24,25,26]. Of particular interest to the current investigation, Klonsky and May [27] observed differences across UPPS subscales between individuals with suicidal ideation and those who attempted suicide. Moreover, Klonsky and May [27] found that only individuals who had attempted suicide in the past demonstrated (lack of) premeditation. High urgency was observed in both individuals with suicidal ideation and those who had made attempts in the past. Elevated levels of sensation seeking and (lack of) perseverance were not observed within either group. Dvorak, Lamis and Malone [28] provided support for using a four-factor model of impulsivity in college samples. In contrast to studies conducted with adult samples, all factors of impulsivity (including Sensation Seeking) were associated with suicide proneness after controlling for depression.

One study has examined the incremental validity of the UPPS facets on suicidal behavior in a predominantly African American sample. Lynam and Colleagues [29] found support for the predictive validity of the UPPS for suicidal behaviors in a sample of individuals receiving inpatient substance abuse treatment. More specifically, urgency and (lack of) premeditation were associated with suicidal behavior; whereas, sensation seeking and (lack of) perseverance did not predict suicidal behavior [29]. However, other studies have documented the role of sensation seeking in predicating suicidal outcomes [28,30]. Additionally, Anestis and Joiner [31] highlighted the amplifying role of urgency, which may further increase the likelihood of someone resorting to suicidal behavior. Taken together, these findings highlight the importance of exploring the incremental validity of the UPPS and how the UPPS facets interact with potential protective factors in reducing risk for suicidal ideation among African American youth.

### 1.2. Reasons for Living as a Mitigating Factor: Protection

Reasons for living stem from the cognitive-behavioral theory of suicidality and posits that suicidal individuals are often less hopeful about the future, and less likely to identify favorable coping resources and life circumstances from which to derive meaning compared to nonsuicidal individuals [32]. Reasons for living have been identified as a suicide protective factor that may decrease the likelihood of suicidality [32,33,34,35,36]. Reasons for living acts as a protective factor against suicidal action in that it may help to decrease impulsive suicidal urges (see [37]). In accordance with this proposition, individuals who report experiencing suicidal urges report a modulating effect of reasons for living on these urges [37,38]. One way reasons for living may impact impulsive suicidal urges is by increasing positive expectations for the future (decreased hopelessness) (see [39]) which may in turn decrease levels of impulsivity [39]. Moreover, previous research has noted that African Americans who report higher levels of reasons for living also report greater levels of social support, and stronger ties to cultural values and racial identity [40,41,42,43]. Therefore, reasons for living may serve as a culturally salient buffer against suicidality among African Americans.

Reasons for living have been found to be associated with suicidal outcomes in community settings, and with outpatient and inpatient populations [32,35,44]. Furthermore, in youth samples taken from undergraduate settings, reasons for living have been found to differentiate individuals who are suicidal and those who are not [45]. A study conducted by Wang and colleagues [19] found that among African American college students, reasons for living had a significant inverse relation to suicidal thoughts and behaviors. However, not all investigations have found an association between reasons for living and suicidality in African American college students [46]. These discrepant findings highlight the importance of continued examination of reasons for living in ethnically diverse populations to further our understanding of how reasons for living contributes to the development, course and maintenance of suicidal outcomes.

### 1.3. The Current Study

Personality constructs (*i.e.*, impulsivity) and protective factors (*i.e.*, reasons for living) have both been used to assess suicidality among African American populations (e.g., [5,23,29,37]). Thus, the purpose of the current study was to determine the incremental validity of each facet of the UPPS with reason’s for living acting as a protective factor for suicidal ideation. Based on previous research demonstrating an association between impulsivity and suicidal outcomes [23,47], we expected to find an interaction between the different UPPS facets and reasons for living on suicidal ideation. However, given that the literature linking the impulsivity facets to suicidal outcomes is somewhat unclear, we propose exploratory hypotheses for the interaction between reasons for living and each of the UPPS facets on suicidal ideation. Specifically, we hypothesize that each facet of the UPPS (*i.e.*, urgency, perseverance, premeditation and sensation seeking) would significantly interact with reasons for living and uniquely contribute to suicidal ideation.

## 2. Methods

### 2.1. Participants

Data were collected from 1,101 undergraduate psychology students at a large southeastern university. For the purposes of the current study, only African American students (*N* =130) were included. Participants were between the ages of 18 and 24 years (*M* age = 19.74, *SD* = 1.60), and 74.6% (*n* = 97) were female. The sample consisted of freshmen (*n* = 54, 41.5%), sophomores (*n* = 19, 14.6%), juniors (*n* = 29, 22.3%) and seniors (*n* = 28, 21.5%). Eighty-one (62.3%) of the students reported they were not in a relationship, and 88.5% (*n* = 115) reported living on campus. Of the students who participated in the study, 8.5% (*n* = 11) indicated that they were a member of a social fraternity or sorority.

### 2.2. Measures

#### 2.2.1. Covariates

Given that undergraduate students are a heterogeneous sample who have met different developmental milestones and who are at various stages of development [48], we controlled for potential variables that may differentiate individuals in our sample, thus, potentially influencing the results. Thus, variables such as age, gender, living situation (on campus/off campus), sorority/fraternity affiliation (yes/no), class in school, and relationship status (not in a relationship *vs*. in a relationship) were controlled for in all analyses. Moreover, given that suicide ideation has been found to be associated with social desirability [49], social support [50,51], and depressive symptoms [52,53], these constructs were also entered into the model as covariates.

*The Marlowe-Crowne Social Desirability Scale-Form B* (MCSD-B; [54]) was used to measure the tendency of making socially desirable responses. The MCSD-B consists of 12 dichotomously scored (true/false) items and was developed from the original Marlowe-Crowne Social Desirability Scale [55]. Sample items include “No matter who I’m talking to, I’m always a good listener” and “I have never deliberately said something that hurt someone’s feelings.” Previous research (e.g., [56]) regarding the MCSD-B has demonstrated adequate internal consistency and validity. The internal consistency estimate in the current sample was 0.69.

*The Multidimensional Scale of Perceived Social Support* (MSPSS; [57]) is a 12-item self-report instrument assessing perceived social support from family, friends, and significant others. The authors characterize the MSPSS as easy to administer and ideal in research settings. Sample items include “My friends really try to help me” and “I get emotional help and support from my family.” Response options range from 1 (*very strongly disagree*) to 7 (*very strongly agree*), with higher scores indicating higher levels of global satisfaction with perceived support. Recently, Osman, Lamis, Freedenthal, Gutierrez, and McNaughton-Cassill [58] found support for the use of the MSPSS as a unidimensional instrument, and the MSPSS has demonstrated solid psychometric properties in university student samples [59,60]. In the current study, the Cronbach alpha for the MSPSS was 0.96.

*The Beck Depression Inventory-II* (BDI-II, [61]) is a widely used 21-item self-report measure of the severity of depressive symptoms. The items (groups of specific statements) are scored from 0 to 3 to assess an individual’s level of symptom severity, with a total score ranging from 0 to 63, which is indicative of high depressive symptomatology. An example of an item on the BDI-II is “Sadness”, with response options being 0 (*I do not feel sad*), 1 (*I feel sad much of the time*), 2 (*I am sad all of the time*), and 3 (*I am so sad or unhappy that I can’t stand it*). Good estimates of internal consistency and concurrent validity have been demonstrated in African American college students [62,63]. In the current study, the internal consistency reliability estimate was 0.92.

#### 2.2.2. Independent Variables

The UPPS Impulsive Behavior Scale [20] (UPPS) is a 45-item measure assessing four facets of impulsive behavior. Participants respond to statements on a scale ranging from 1 (strongly agree) to 4 (strongly disagree), with higher scores indicating more impulsivity in each subscale. The four facets include urgency (12 items, α = 0.87, example: “I have trouble controlling my impulses”), (lack of) premeditation (11 items, α = 0.86, example: “I have a reserved and cautious attitude toward life”), (lack of) perseverance (10 items, α = 0.72, example: “I generally like to see things through to the end”), and sensation seeking (12 items, α = 0.79, example: “I generally seek new and exciting experiences and sensations”). Researchers (e.g., [64] have demonstrated good psychometric properties for the UPPS Impulsive Behavior Scale, and it has been successfully used in college student samples [27,28].

*The* *Reasons for Living Inventory for Young Adults* (RFL-YA; [65] is composed of 32-items that are constructed to assess reasons young adults give for not engaging in suicide-related behaviors (*i.e.*, reasons for living). Each item is rated on a 6-point scale ranging from 1 (*not at all* *important*) to 6 (*extremely important*). Sample items of the RFL-YA include “I have a close relationship with my family” and “my friends stand by me whenever I have a problem” [65]. In the current study, responses on the items were averaged to obtain a total scale score, with higher scores being indicative of stronger reasons for living. The use of the total RFL score has been used in previous studies with college students [66,67]. Estimates of internal consistency and concurrent validity of the total RFL-YA have been demonstrated to be satisfactory in young adults [68]. The internal consistency reliability estimate in the present study was 0.98.

#### 2.2.3. Dependent Variable

*The Modified Scale for Suicide Ideation* (MSSI; [69]) is an 18-item self-report continuous measure that assesses the presence and severity of suicidal ideation during the past two weeks. Participants rated each item on a 4-point scale, which were summed to obtain a total scale score ranging from 0 to 54, with higher scores indicating higher levels of suicidal ideation. For example, response options for the sample question are “0 = *I have not thought about how I would kill myself*; 1 = *I have thought about it, but I was not sure how*; 2 = *I knew how I wanted to kill myself, but the details were not clear*; or 3 = *I knew exactly how I wanted to kill myself*.” Higher scores on the MSSI have been found to be significantly correlated with greater hopelessness, depression, and measures of suicidal thoughts and behaviors [70]. The MSSI has been successfully used in samples of college students [71] and has demonstrated good estimates of internal consistency, convergent validity, and discriminant validity in studies with clinical and non-clinical samples [72,73]. The Cronbach alpha in the current sample was 0.96.

### 2.3. Procedure

All subjects gave their informed consent for inclusion before they participated in the study. The study was conducted in accordance with the university’s Institutional Review Board protocol (IRB registration number: 00000240). The university’s Institutional Review Board approved the study in advance of data collection, and ethical guidelines for data collection were followed throughout the study. Using a secure on-line survey procedure, we collected data over the course of two consecutive academic semesters, with approximately equal numbers of participants completing the study during each of the semesters. Information regarding the study was announced in several regularly scheduled classes and through a posting on the online participant pool site. In return for extra credit in their psychology course, all participants provided written informed consent before voluntarily completing the questionnaires, which were presented in a randomized order. Potential participants were advised that some items in the survey were personal in nature and that all demographic information regarding participants would anonymous. Participants were also advised that they were free to leave any item blank in each questionnaire.

## 3. Results

### 3.1. Data Screening

Given that the suicide ideation variable measured with the MSSI displayed substantial positive skew and kurtosis (skewness = 3.68; kurtosis = 12.55), these scores were transformed using a natural log transformation (plus two). Although the skewness and kurtosis did not reach conventional values [74], the log transformation decreased skewness (2.66) and kurtosis (6.53) allowing our distribution to approach normality. For greater interpretability of the subsequent analyses, given that the log transformed MSSI scores showed the same pattern of effects as the original data, all analyses were conducted using the original MSSI variable and not the log transformed MSSI variable.

### 3.2. Descriptive Statistics

Means, standard deviations and intercorrelations for all primary study measures (*i.e.*, impulsive behavior scale, reasons for living and suicide ideation) are presented in Table 1. Partial correlations of these measures after controlling for age, gender, living situation, sorority/fraternity affiliation, class in school, relationship status, social desirability, social support, and depressive symptoms are also presented. Suicidal ideation was correlated with reasons for living and the UPPS (lack of) premeditation facet after controlling for study covariates. Reasons for living were also correlated with all measures of the UPPS except for the sensation seeking facet. However, after controlling for study covariates, the reasons for living scale did not correlate with the UPPS urgency facet. The mean level of the MSSI fell in the low range (*M* = 2.15, *SD* = 6.50), suggesting that our sample of participants were experiencing minimal levels of suicidal ideation. Scores on the MSSI ranged from 0 to 32. Approximately 5.4% of the sample reported severe levels of suicidal ideation (*i.e.*, scores above 21), 1.6% reported mild to moderate suicidal ideation (*i.e.*, scores between 9 and 20), and 93.1% reported low levels of suicidal ideation (*i.e.*, scores between 0 and 8).

### 3.3. Incremental Validity of the UPPS

To test the incremental predictive utility of the UPPS facets, we conducted hierarchical regression analyses with each UPPS facet interacting with reasons for living in association with suicidal ideation, see Table 2. The dependent variable in these analyses was the MSSI (suicide ideation) scores. Six hierarchical regression analyses were examined for the covariates model, the main effects model and the models with each of the reasons for living by UPPS facets. Each interaction was entered into a separate model so that the interaction terms did not compete with one another and to reduce multicollinearity. In the first step of each regression equation (model 1 through 6 on Table 1), the covariates (*i.e.*, age, gender, living situation, sorority/fraternity affiliation, class in school, relationship status, social desirability, social support, and depressive symptoms) were entered. In the second step of the regression equation (model 2 through 6 on Table 1), the main effects of self-reported reasons for living, and each UPPS facet (*i.e.*, urgency, premeditation, perseverance and sensation seeking) were entered. Results revealed that the main effects of reasons for living, (lack of) perseverance and (lack of) premeditation, but not urgency or sensation seeking were significantly associated with suicidal ideation (model 2 on Table 1). The inclusion of these predictor variables accounted for 19% of the variance [∆*F* (5, 115) = 7.20, *p* < 0.001] in the model beyond what was accounted for by the covariates.

**Table 1:** Means, Standard Deviations, and Intercorrelations for All Measures.

Variables	1	2	3	4	5	6
1. MSSI	--	−0.55 **	0.32 **	0.16	0.11	0.17
2. RFL	−0.43 **	--	−0.42 **	−0.17 *	−0.11	−0.43 **
3. PREM	0.27 **	−0.38 **	--	0.27 **	0.13	0.60 **
4. URG	0.06	−0.04	0.17	--	0.22 *	0.42 **
5. SS	0.10	−0.02	0.09	0.20 *	--	0.13
6. PER	0.03	−0.29 **	0.56 **	0.31 **	0.09	--
*M*	2.15	5.33	18.48	26.40	30.44	19.45
*SD*	6.50	87	5.24	7.11	6.51	4.08

*N* = 130. Zero-order correlations (above the diagonal) and partial correlations (below the diagonal) after controlling for Age, gender, living situation, sorority/fraternity affiliation, class in school, relationship status, social desirability, social support, and depressive symptoms. MSSI = The Modified Scale for Suicide Ideation. RFL = The Reasons for Living Inventory for Young Adults. PREM = The UPPS Impulsive Behavior Scale (Premeditation facet). URG = The UPPS Impulsive Behavior Scale (Urgency facet). SS = The UPPS Impulsive Behavior Scale (Sensation Seeking facet). PER = The UPPS Impulsive Behavior Scale (Perseverance facet). *p < 0.05, **p < 0.01.

**Figure 1:**
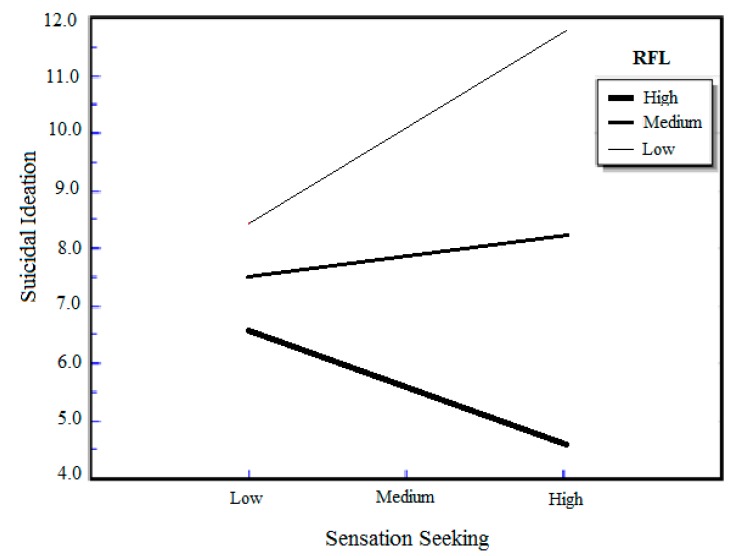
Association between sensation seeking and suicidal ideation at three levels of reasosn for living (RFL).

**Figure 2:**
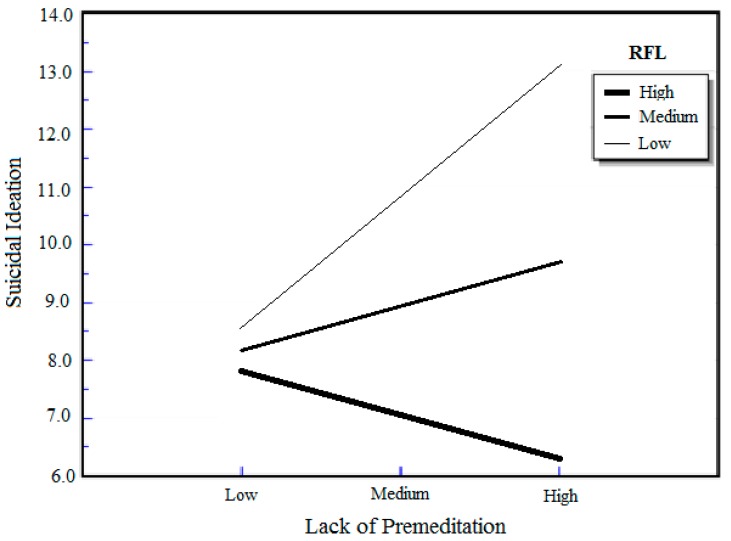
Association between lack of premeditation and suicidal ideation at three levels of reasons for living (RFL).

The reasons for living by UPPS facets interaction terms were entered in the third step of each equation (*i.e.*, reasons for living by urgency, reasons for living by (lack of) premeditation, reasons for living by sensation seeking, and reasons for living by (lack of) perseverance). Mean-centering was conducted in order to ensure that the coefficient for the interaction variables will be interpretable based on the range of values in the data [75]. As seen in Table 2, the interaction between reasons for living and the UPPS sensation seeking facet in model 3 (*b* = −0.23, *p* = 0.03) and the interaction between reasons for living and the UPPS (lack of) premeditation facet in model 4 (*b* = −0.33, *p* < 0.001) were significantly associated with suicidal ideation. The inclusion of the UPPS by sensation seeking facet account for an additional 2.6% [∆*F* (1, 114) = 5.02, *p* = 0.03] of variance on suicidal ideation above what was accounted for by the covariates and main effects. The unstandardized simple slope for sensation seeking predicting suicide ideation at 1 *SD* above the mean of reasons for living was −0.15 (*p* = 0.39), the unstandardized simple slope for sensation seeking with a mean level of reasons for living was 0.05 (*p* = 0.24), and the unstandardized simple slope for sensation seeking at 1 *SD* below the mean of reasons for living was 0.25 (*p* = 0.31) (see Figure 1). The inclusion of the UPPS by (lack of) premeditation facet accounted for 6.8% [∆*F* (1, 114) = 14.45, *p* < 0.001] of the variance on suicidal ideation above what was accounted for by the covariates and main effects. The unstandardized simple slope for lack of premeditation predicting suicide ideation at 1 SD above the mean of reasons for living was −3.94 (*p* = 0.09), the unstandardized simple slope for lack of premeditation with a mean level of reasons for living was −2.19 (*p* < 0.001), and the unstandardized simple slope for lack of premeditation at 1 *SD* below the mean of reasons for living was −0.44 (*p* = 0.45) (see Figure 2). Figure 1 and Figure 2 show a graphic illustration of the interaction between reasons for living and sensation seeking, and the interaction between reasons for living and (lack of) premeditation, respectively. The interaction between reasons for living and the UPPS urgency facet (*b* = −0.13, *p* = 0.18) in model 5 of Table 2, and the interaction between reasons for living and the UPPS (lack of) perseverance facet (*b* = −0.06, *p* = 0.57) in model 6 of Table 2 were not significantly associated with suicidal ideation.

**Table 2:** Summary of Hierarchical Regression Analyses for Variables Predicting Suicidal Ideation.

Variables	Model 1	Model 2	Model 3	Model 4	Model 5	Model 6
Covariates						
Gen	2.63 *	1.23	1.00	1.19	1.21	1.35
Age	−0.56	−0.36	−0.41	−0.43	−0.43	−0.41
Relstat	−1.19	−1.53	−1.40	−1.47	−1.54	−1.45
Class	0.48	0.18	0.27	0.03	0.27	0.19
Reside	0.11	0.32	0.30	0.36	0.30	0.35
Club	1.55	1.08	1.88	0.70	0.99	1.08
MSPSS	−0.07 *	−0.02	−0.03	−0.02	−0.02	−0.02
SOCDES	0.25	0.19	0.21	0.15	0.19	0.08
BDI	0.26 **	0.14 *	0.15 *	0.16 *	0.14 *	0.15 *
Predictors						
RFL	--	−3.09 **	−2.60 **	−2.20 **	−3.16 **	−3.09 **
SS	--	0.03	0.05	0.04	0.03	0.02
PREM	--	0.29 *	0.33 **	0.14	0.26 *	0.31 *
URG	--	0.06	0.09	0.00	0.09	0.06
PERS	--	−0.40 *	−0.46 **	−0.22	−0.42 *	−0.43 *
Interaction Terms						
RFL X SS	--	--	−0.23 *	--	--	--
RFL X PREM	--	--	--	−0.33**	--	--
RFL X URG	--	--	--	--	−0.13	--
RFL X PERS	--	--	--	--	--	−0.06
∆R²	0.20	0.19	0.03	0.07	0.01	0.00

All entries are presented with standardized betas. All predictor variables for the interaction terms are centered. Gen = gender. Relstat = relationship status. Class = class in school. Reside = living situation. Club = sorority/fraternity affiliation. MSPSS = The Multidimensional Scale of Perceived Social Support. SOCDES = The Marlowe-Crowne Social Desirability Scale-Form B. BDI = Beck Depression Inventory-II. RFL = The Reasons for Living Inventory for Young Adults. SS = The UPPS Impulsive Behavior Scale (Sensation Seeking facet). PREM = The UPPS Impulsive Behavior Scale (Premeditation facet). URG = The UPPS Impulsive Behavior Scale (Urgency facet). PERS = The UPPS Impulsive Behavior Scale (Perseverance facet). * *p* < 0.05, ** *p* < 0.01.

## 4. Discussion and Conclusions

Using a framework that examines both risk and protective factors for understanding psychopathology (see [9,10,11]), we aimed to determine the incremental validity of each facet of the UPPS, a well-known and used measure of impulsivity, and their interaction with reasons for living in the association with suicidal ideation in a sample of African American young adults. Prior to examining the incremental validity of the UPPS, preliminary analyses revealed an association between reasons for living and suicidal ideation. Specifically, more reasons for living were associated with lower suicidal ideation. This is consistent with previous studies showing a relation between reasons for living and suicidality in African American populations (e.g., [36,37,38,41,47]). However, previous research regarding reasons for living and suicidal ideation in African American youth has been inconsistent. For example, Morrison and Downey [5] examined race group differences with reasons for living and found that African American college students scored higher on the reasons for living inventory, particularly on areas of moral objections against suicide than their European American study counterparts. African Americans also reported utilizing higher levels of effective coping strategies that helped minimize suicidality than the European American sample. Contrary to this finding, an investigation by Marion and Range [43] failed to find a significant association between reasons for living and suicidality in a sample of African American college students. This discrepancy notwithstanding, the result of our current analysis adds to the growing body of research showing a connection between reasons for living and suicidality among African American populations.

We found partial support for the primary aim in the present investigation, which sought to examine the interaction between reasons for living and the different impulsivity facets on suicidal ideation. Only some facets of impulsivity interacted with reasons for living in association with suicidal ideation; outlining the differential association of impulsivity on suicidal ideation. A significant interaction effect of reasons for living and lack of premeditation was found, such that lower levels of reasons for living and higher levels of lack of premeditation were associated with greater suicidal ideation. These significant effects were found even when depressive symptoms and other relevant covariates were controlled for in our current sample of African American youth. Lack of premeditation emerged as both a robust main effect as well as interaction effect measure of impulsivity in assessing suicidal ideation, indicating particular usefulness of this facet. These findings are consistent with previous investigations that have also found an association between lack of premeditation and suicidality [27,29], signaling the need for intervention protocols to increase the level of forethought (premeditation) as a means of mitigating suicidal outcomes among high risk youth.

Our results revealed a significant interaction between reasons for living and sensation seeking on suicidal ideation, such that higher levels of sensation seeking and lower levels of reasons for living were associated with increased suicidal ideation. Previous investigations assessing the link between sensation seeking and suicidality have been somewhat inconsistent, with some studies not finding significant associations [27,29], whereas, others have demonstrated a relation between the experience of sensation seeking and suicidal outcomes in young adult populations [30,76]. Studies that highlight a link between sensation seeking and suicidal outcomes often describe suicidality as a risky behavior much like substance use and reckless driving, and thus emphasize similar pathways from sensation seeking to suicidal outcomes as those found with other risky behaviors (see [30]).

Another possible explanation for the significant association found with lower levels of reasons for living and sensation seeking on suicidal ideation is that the significant effect found with the sensation seeking facet might be accounted for by other potential covariates not partialed out from the current model. In a recent study, Ortin and colleagues [30] found an effect of sensation seeking on suicidal ideation and suicide attempts. They proposed that these significant effects might be understood through an underlying temperamental dysregulation found among those experiencing subclinical levels of psychopathology. These investigators proposed that the high levels of sensations seeking and suicidal ideation found in some individuals may be a potential marker for early stages of bipolarity (see [77,78]). Although these propositions go beyond the scope of the current investigation, future researchers assessing suicidality would benefit from delineating potential mechanisms by which sensation seeking may influence suicidal ideation in African American youth. Furthermore, given the exploratory nature of our analyses, future research would benefit from replicating these findings in similar age ranged samples and other African American sub-populations to assess the generalizability of these findings.

Neither the negative urgency nor the lack of perseverance facets interacted with reasons for living in association with suicidal ideation. Previous reports on (lack of) perseverance on suicidality have been consistent with the current findings, showing no association between (lack of) perseverance and suicidal outcomes [27,29]. Thus, lack of perseverance may not be as relevant with respect to predicting and understanding the development of suicidal outcomes as other measures of impulsivity. Given the role of negative affect in self-destructive behaviors [27,29], urgency, which is a tendency to act impulsively during states of negative affect may thus relate to suicidality. However, we did not find a significant interaction between reasons for living by urgency on suicidal ideation. Although surprising, previous investigations have found that urgency may not be independently associated with suicidality [29]. Therefore, negative urgency may be a more distal factor that can become particularly influential in the face of other markers of distress. These findings need to be replicated to determine if they are generalizable to similar samples and other populations.

Though the present study is useful in helping to delineate the facets of impulsivity that are most relevant when assessing for suicidal ideation in the face of limited reasons for living, the study limitations must be acknowledged. First, the cross-sectional design of this study precludes our ability to draw causal conclusions. Accordingly, prospective examination of the variables of interest would extend the current research. Second, this model assesses suicidal ideation and does not differentiate between active and passive ideation. Thus, future studies would benefit from examining different descriptions of suicidality with the inclusion of suicidal behavior. Third, our sample consisted of a university sample of African Americans, which may not generalize to other populations. Although those who attend university have unique stressors that may confer risk to suicidal ideation and potential suicidal behavior, these individuals may not experience the same types of socioeconomic, psychological, and marginalized difficulties that those in the general community face. Thus, in order to extend the generalizability of the current model, future research would benefit from examining these constructs with community dwelling adults or with clinical samples of African Americans. Fourth, although we found significant effects in our study, these effects were relatively small which may be explained by the small sample size of the study. In order to address this shortcoming, future research would need to utilize larger samples of participants and incorporate other relevant mediator and moderator variables into the model to derive a comprehensive model of risk and protection.

The present study is noteworthy in that it extends the current body of research on risk and protective factors that contribute to suicidality in African Americans. Assessing reasons for living continues to show promise as a protective factor for African American populations. Therefore, interventions that increase reasons for living in those at risk of suicidality may potentially serve to mitigate negative outcomes. Furthermore, given the effects found for sensation seeking and (lack of) premeditation on suicidal ideation, these personality facets should be particularly emphasized with suicide screens and interventions for young adult populations with low levels of reasons for living. Therapeutic strategies such as dialectical behavior therapy (DBT; [79]) which focus on alleviating distress through client awareness of their emotional state and building of skills will be helpful in reducing suicidal outcomes. DBT may be potentially helpful in enhancing protective factors for clients and reducing levels of impulsivity by increasing levels of emotion awareness, forethought, mindfulness and self-regulatory practices (see [80]). Consistent with this notion, DBT-skills based programs have been found to improve impulsive behaviors [81,82,83,84] and decrease levels of suicidality (e.g., [76,85,86]). Further, investigations assessing factors that influence the onset and course of suicidal ideation should make note of the complexity of suicidality. Suicide cannot be fully understood with deficit models. Examining the development of suicidality using models of risk and protection, and with attention to salient cultural variables broadens our understanding of factors that help develop, maintain, and mitigate the development of suicidal ideation.
